# Visuospatial experience shapes the form of gestures: Blind speakers gesture with more precise spatial tracking of motion than sighted speakers

**DOI:** 10.3758/s13423-026-02957-w

**Published:** 2026-07-29

**Authors:** Ezgi Mamus, Mounika Kanakanti, Sho Akamine, Aslı Özyürek

**Affiliations:** 1 Max Planck Institute for Psycholinguistics, Wundtlaan 1, 6525XD Nijmegen, The Netherlands; 2 Donders Center for Cognition, Radboud University, Nijmegen, The Netherlands

**Keywords:** Gesture kinematics, Blind, Visual experience, Path gestures, Spatial language, Motion tracking

## Abstract

Co-speech gestures emerge from the interaction between visuospatial experience and speech formulation, but what happens when visual experience is absent from birth? Blind individuals gesture in ways consistent with their native language, albeit they gesture less frequently. However, nothing is known about whether their gesture form differs from that of sighted individuals. Using computer vision technologies, this study examines whether gesture kinematics are shaped by visuospatial experience. In an auditory motion-description task, we analyzed spontaneous path-only gestures – depicting motion trajectories – produced by 20 congenitally blind, 21 blindfolded, and 21 sighted Turkish speakers. We assessed the gestures’ alignment with actual motion paths, along with duration and size. Blind participants produced gestures that were more precisely aligned with the motion trajectories, as well as larger and longer. These findings show that altered spatial cognition in blindness enhances gesture precision. The study underscores the role of sensorimotor experience in shaping gesture form.

## Introduction

All speakers gesture as they speak (Kita et al., 2017; McNeill, 1992). These co-speech gestures, especially those that have iconic properties (e.g., depicting how big something is or how it moves), systematically vary across languages, mirroring the language-specific grammatical structure (Kita & Özyürek, 2003). Remarkably, individuals who are blind from birth also gesture in language-specific ways, suggesting that language alone is enough for developing native-like gestural behavior (Özçalışkan et al., 2016, 2018). Nevertheless, blind speakers produce fewer spontaneous iconic gestures than sighted speakers (Iverson, 1999; Iverson & Goldin-Meadow, 1997; Mamus et al., 2023). To date, most research has focused on the frequency and grammatical properties of gestures produced by blind speakers, leaving open the question of whether variations in their visuospatial experience influence the form of their gestures.

Form of iconic gestures – such as size and trajectory – reflects how individuals interact with objects and events (Beilock & Goldin-Meadow, 2010; Cook & Tanenhaus, 2009; Pouw et al., 2020). Several theories therefore propose that gestures are shaped not only by how speech is formulated but also by speakers’ visuospatial and motor experience (Hostetter & Alibali, 2008; Kita et al., 2017; Kita & Özyürek, 2003). Thus, blind speakers’ distinct visuospatial experience may influence the form of their gestures. Recent developments in computer vision and motion-tracking technologies now enable more fine-grained analysis of the form characteristics of gestures. Using these tools, the present study goes beyond traditional quantitative measures and investigates whether gesture kinematics are shaped differently in blindness. By introducing a novel method to measure gesture precision, this research offers fresh insights into the cognitive processes underlying gesture production and opens up new directions for studying human communication in the era of artificial intelligence.

We address this question by examining whether the spatial placement and trajectory of gestures accompanying verbal path descriptions reflect the distinct visuospatial experience of blind people. Prior research shows that blind people outperform sighted and blindfolded people in sound localization and detecting movement changes, thus demonstrating an enhanced ability to track motion auditorily (Battal et al., 2020; Dufour et al., 2005; Lessard et al., 1998; Nilsson & Schenkman, 2016; Röder et al., 1999). Given that co-speech gestures are also shaped by individuals’ visuospatial and motor experience (Hostetter & Alibali, 2008), these enhanced spatial abilities of blind people may influence the kinematic features of their gestures – particularly how precisely motion paths are represented during event descriptions.

Language studies have also shown that blind people’s language use can reflect features of their particular experience of space (Iverson, 1999; Iverson & Goldin-Meadow, 1997; Mamus et al., 2023). Blind people experience large-scale environments through kinesthetic, auditory, and haptic cues, which are processed sequentially in contrast to the holistic nature of visual information, making it challenging to construct map-like spatial representations (Cattaneo & Vecchi, 2011; Thinus-Blanc & Gaunet, 1997). As a result, blind people often adopt an egocentric perspective – relying on their own body’s perspective and position – and build representations anchored by landmarks (Coluccia et al., 2009; Iachini et al., 2014; Pasqualotto et al., 2013; Ruggiero et al., 2021). This change in spatial cognition is reflected in how they talk about spatial events. For example, they segment paths using multiple landmarks when describing familiar routes (Iverson, 1999; Iverson & Goldin-Meadow, 1997). Blind speakers also describe landmarks relative to their own position in space (e.g., “Someone got out of the elevator from my left”), which is rarely observed among sighted and blindfolded speakers (Mamus et al., 2023). Together, these results propose that blind people’s spatial language reveals particular features of their unique spatial representations, supporting theories that posit a close link between perception and language (Bryant, 1997; Landau & Jackendoff, 1993; Talmy, 1983; Taylor & Tversky, 1992). However, whether blindness similarly shapes the form of gestures remains unexplored.

Theories of co-speech gesture production posit that gestures are shaped, to some extent, by what is expressed in speech (Kita & Özyürek, 2003). For example, when describing motion events, English speakers typically express path and manner components in a single clause (e.g., she ran into the house), with manner as the main verb. In contrast, speakers of Turkish and Japanese express path in the main verb and encodes manner optionally through subordinated verbs or adverbial phrases (e.g., *koşarak eve girdi*, “she entered the house running”) (Slobin, 1996; Talmy, 1985). A substantial body of research shows that co-speech gestures often mirror these cross-linguistic patterns in motion-event encoding (e.g., Gullberg et al., 2008; see Ünal et al., 2024, for a review). English speakers produce gestures that conflate path and manner, whereas Turkish and Japanese speakers gesture them separately (Kita & Özyürek, 2003). Notably, similar trends have also been observed in comparisons of blind and sighted Turkish and English speakers’ gestures, suggesting that blind people’s gestures also mirror the specific linguistic structure (Mamus et al., 2023; Özçalışkan et al., 2016).

Although gesture production interacts with speech production during the planning and execution stages (Chu & Hagoort, 2014; Kita & Özyürek, 2003; Ünal et al., 2022), a core argument across different models of gesture production is that gestures – particularly iconic gestures – also arise from visuospatial and motor experiences (Hostetter & Alibali, 2008; Kita et al., 2017; Kita & Özyürek, 2003). Gesture forms reflect gesturers’ specific sensorimotor experiences with objects and events (Beilock & Goldin-Meadow, 2010; Cook & Tanenhaus, 2009; Pouw et al., 2020). These findings raise the important question of whether the gesture forms of blind speakers are also shaped by their enhanced spatial abilities in tracking motion (Battal et al., 2020; Lessard et al., 1998; Röder et al., 1999), in addition to language-specific representations. Gestures depicting paths in motion events provide a good testbed for addressing this question, a domain that reflects language specificity and yet may be shaped by cognition of blind individuals.

### Current study

The present study examines whether blind speakers’ path gestures are more precise than those of blindfolded and sighted speakers, reflecting their enhanced ability to track motion experienced auditorily. To this end, we use computer vision models to examine the kinematic features of path gestures – particularly their spatial precision – spontaneously produced by blind, blindfolded, and sighted speakers during motion-event descriptions. By tracking hand movements during gesturing, we compute the angular precision of path gestures that depict the motion trajectory.

Previous research on gesture kinematics has focused on features such as size, amplitude, velocity, and submovement structure to understand the complexity and informativeness of gesture production (Pouw et al., 2021; Trujillo et al., 2018). These kinematic properties are often modulated for audience design, reflecting speakers’ efforts to signal communicative intent and highlight information relevant to the addressee (Trujillo et al., 2018, 2020). For example, gestures produced in more communicative contexts tend to be larger, include more submovements, and exhibit higher peak velocities than gestures produced in less communicative contexts (Trujillo et al., 2018). Similarly, gesture kinematics adapt to noisy conditions, further supporting the view that speakers modulate their gestures according to the communicative context (Trujillo et al., 2021).

Unlike prior research that primarily examined gesture kinematics in communicative versus non-communicative contexts, the present study investigates gestures produced in a relatively less communicative setting (i.e., with no addressee present) to determine whether differences in spatial cognition of blind people influence their gesture kinematics. Accordingly, our primary focus and novel aspect of this study is gesture precision. To further contextualize our claims, we also measure gesture size and duration, as both are closely related to how the motion trajectory is represented during gesturing.

If speakers track motion more precisely, their gestures may depict the continuous trajectory of the movement rather than indicating only its general direction in a more abstract way. Representing an entire trajectory may require tracing a longer path in space, thereby mapping the spatial extent of the motion and resulting in larger gestures. Such larger gestures could also be expected to last longer. At the same time, it is theoretically possible to accurately indicate only the direction of a movement with a brief gesture that points to the final location without tracing the path. In such cases, gesture size would remain small despite accurate localization. For this reason, gesture size and duration serve as complementary measures that help interpret the results of gesture precision.

**Fig. 1:**
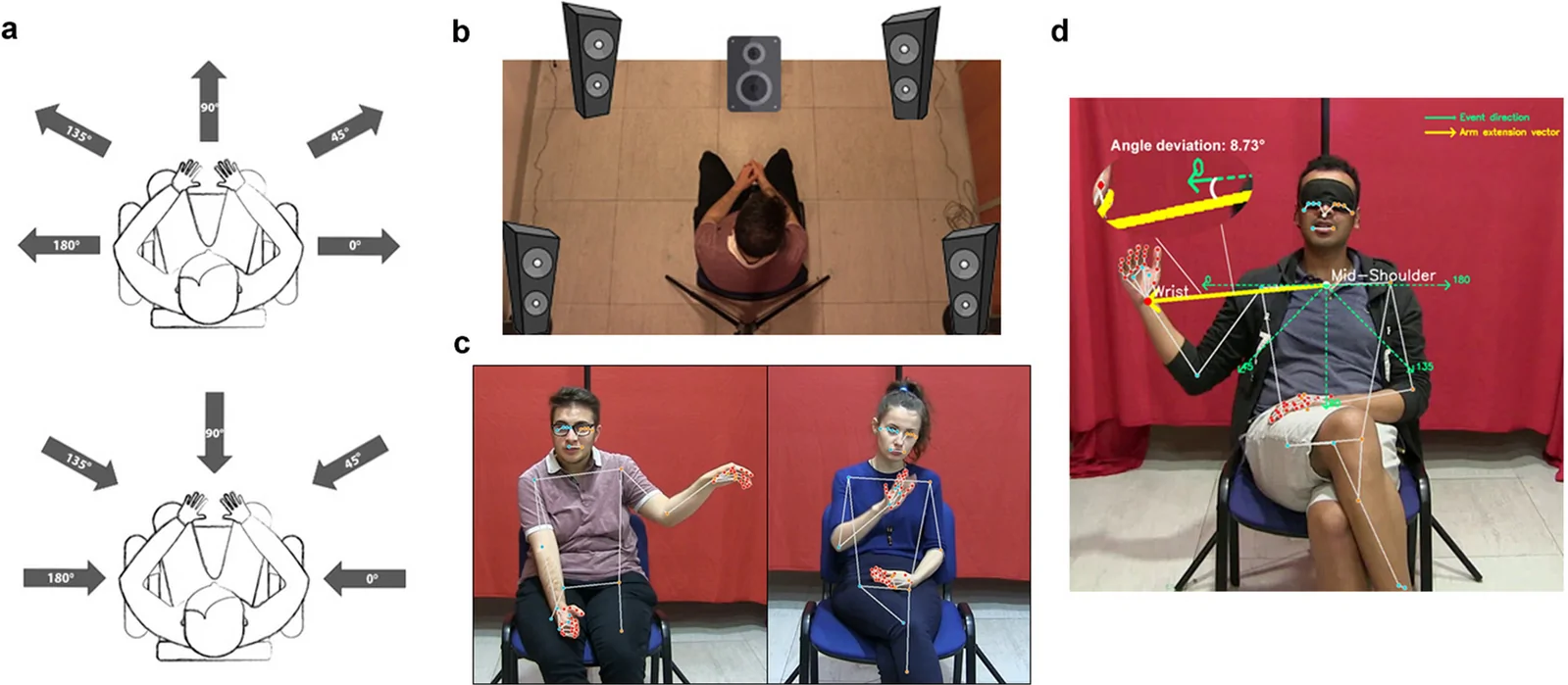
**(a)** Five movement angles for “from“ and “out of“ events (top; motion moving away from participants) and “to“ and “into“ events (bottom; motion approaching participants). **(b)** Experimental setup showing the placement of sound speakers. **(c)** Path gestures with keypoint markers: Left – a blind participant producing a path gesture (hand moving away toward the speakers’ left) while saying “headed towards that way”; Right – a sighted participant producing a path gesture (hand moving across away from the speaker) while saying “walked out the door”. **(d)** An example of gesture direction detection and angular deviation analysis, showing arm extension vector calculation for a blindfolded participant. Angular deviation between the gesture vector and the actual event location was calculated using the dot product. The yellow arrow represents the gesture vector, while green arrows indicate the event locations relative to the participant’s orientation

We analyzed co-speech gesture data from Mamus et al. (2023). They created auditory motion events to equate input modality for all participants and to provide ecologically relevant stimuli, as hearing the sounds of human locomotion is a part of daily life for both blind and sighted people. The direction of the events was also manipulated to increase path diversity. Specifically, the events were presented from five angles in a semicircle, moving toward or away from participants (Fig. 1a).

To quantify the alignment of speakers’ path gestures with the actual spatial location of the events, we calculated their deviation from the event location in angular degrees. We used MediaPipe for motion tracking (Fig. 1c) and developed a computational framework that processes motion capture data. If blind people’s unique spatial cognition influences how they gesture, their gestures should track the motion trajectory more precisely than those of sighted and blindfolded speakers. Furthermore, if blind speakers trace the entire motion trajectory with their path gestures, these gestures should be larger than those of sighted and blindfolded speakers, with correspondingly longer gesture durations.

## Method

### Participants

This study used data from Mamus et al. (2023). A total of 20 congenitally blind individuals (*M*_*age*_ = 28.19 years, *SD* = 6.56, range = 18–40), 21 blindfolded participants (*M*_*age*_ = 27.43 years, *SD* = 6.10, range = 19–49), and 21 sighted individuals (*M*_*age*_ = 27.29 years, *SD* = 6.61, range = 20–41) who were native Turkish speakers participated in the experiment. The sample size of the initial study was determined by access to the special population, with matched control groups, and is comparable to or larger than previous studies involving the same population (Iverson, 1999; Iverson & Goldin-Meadow, 1997; Özçalışkan et al., 2016, 2018). Twelve blind participants had light perception, while eight were completely blind. Blindfolded and sighted participants, all with normal or corrected-to-normal vision, were matched to the blind group based on age, gender, and education. All participants received monetary compensation for their participation. They provided written informed consent approved by Boğaziçi and Radboud Universities’ IRB committees.

### Stimuli and procedure

The auditory motion events were created by recording the sounds of human locomotion (Mamus et al., 2023). An actress performed three different manners (walking, running, and limping) along four paths (to, from, into, and out of) in relation to a landmark object (door or elevator) – for example, “someone walks out of an elevator.” The direction of the events was also manipulated to increase path diversity. The events were presented from five movement angles spanning from 90° left to 90° right, with 45° intervals. These angles, from right to left, were: 0° (right), 45° (right-sided), 90° (front), 135° (left-sided), and 180° (left) (Fig. 1a). The sounds approached or moved away from the participants from these five movement angles. This resulted in 60 events in total, which lasted an average of 9 s (*SD* = 1.9). For more details on the creation of the stimuli, see Mamus et al. (2023).

The procedure was identical for all groups, except that blindfolded participants had their eyes covered with a mask before entering the room. Five speakers were positioned in a 5 + 1 surround-sound configuration (Fig. 1b). The front left and right speakers were placed 30° off-center, while the rear left and right speakers were 110° off-center. Participants sat in the center of the speakers. The events were presented auditorily, and participants were asked to describe each event at their own pace. No instructions regarding gesture use were given.

### Coding and kinematic measures calculation

Manual coding for spontaneously produced gestures was conducted in the earlier study (Mamus et al., 2023). Each gesture stroke (i.e., the meaningful phase of a gesture) was identified and annotated in ELAN. For our purposes, we analyzed only *path-only* gestures; *path+manner* gestures were not included. Path-only gestures represent the movement trajectory without depicting the manner of the motion, and they were the dominant gesture type across all participant groups. Specifically, 99.4% of path-related gestures (defined as path-only and path+manner gestures) in the blind group were *path-only*, compared to 82.6% in the blindfolded group and 90.4% in the sighted group. Thus, our analyses capture the vast majority of path-related gestures in the corpus.

We used MediaPipe for motion tracking (Fig. 1c), utilizing a toolbox (Pouw & Trujillo, 2021). Per gesture stroke, we calculated kinematic features of size and duration based on the wrist position of the gesturing hand. Size was defined as the distance the hand traveled during gesture production, measured in unit of shoulder length (where 1 equals the speaker’s shoulder width), because path gestures typically represent the motion trajectory as a line. Duration was defined as the total time taken for each gesture production, measured in milliseconds.

To determine the extent to which the speakers’ path gestures aligned with the actual spatial location of the events, we calculated their deviation from the event location in angular degrees. We developed a computational framework that processes motion capture data in three stages: (a) Body position/reference frame normalization, (b) gesture direction detection, and (c) angular deviation analysis.

#### Body position/reference frame normalization

To make measurements consistent regardless of camera positions and participants’ orientation, we established a body-centered coordinate system that creates a standardized reference frame for each participant. This process used anatomical landmarks to create a reference frame with its origin at the midpoint between the shoulders, the Y-axis aligned with the spine (computed from the shoulder-hip vector), the Z-axis perpendicular to the shoulder line (points forward to the participant’s body), and the X-axis derived from their cross product (points to the participant’s right). All motion capture data was transformed into this normalized reference frame. This process ensures that measurements are comparable across different recordings and camera angles.

#### Gesture direction detection

Within this transformed coordinate space, we computed three distinct vectors to characterize gestural movements:

1. A pointing vector defined from the wrist to the index fingertip,
2. A motion vector calculated from wrist displacement across frame windows, and
3. An arm extension vector measured from the shoulder center to the wrist (see Fig. 1d for an example).

The gesturing hand was automatically determined by analyzing cumulative wrist displacement throughout the gesture sequence.

#### Angular deviation analysis

Event locations were transformed into the participant’s reference frame to ensure that the event locations were represented relative to the participant's orientation, where 0 degrees indicated rightward direction, 90 degrees forward, and 180 degrees leftward (see also Fig. 1a). We computed the angular deviation between the event location and each of the three gesture vectors using the dot product method (Fig. 1d), where v_gesture and v_event represent the normalized direction vectors:

$$\mathrm{Angel}\ \uptheta =\mathrm{arccos}\left({\mathrm{v}}_{\mathrm{gesture}\cdot}\ {\mathrm{v}}_{\mathrm{event}}\right)$$

The minimum angular deviation across all three measures was used as the primary metric of gesture-pointing accuracy, providing a robust measure that accounted for different gestural strategies participants might employ.

## Results

We used linear mixed-effects regression models (Baayen et al., 2008) with the fixed factor of visual status (blind, blindfolded, and sighted) together with random intercepts for participants and items. Models were implemented in R (Version 4.5.1; R Core Team, 2025) using the *lme4* package (Version 1.1–37; Bates et al., 2015) with the *nloptwrap* optimizer, and *p*-values were obtained via *lmerTest* (Version 3.1–3; Kuznetsova et al., 2017). Dependent variables – angle precision, gesture size, and duration – were log-transformed. Model diagnostics and posterior predictive checks confirmed a good fit to the data. To assess statistical significance of the fixed factor, we used likelihood-ratio tests with χ^2^, comparing models with and without the fixed factor. For post hoc pairwise comparisons, we used *emmeans* with the Tukey adjustment (Version 1.11.2; Lenth, 2022). To control for multiple testing across all contrasts in the study, we further adjusted *p*-values using the Benjamini–Hochberg procedure (FDR). Data, scripts, and analysis code are available at: https://osf.io/97skh.

We first present the results for gesture precision, followed by the results for gesture size and duration.

### Gesture precision

**Fig. 2:**
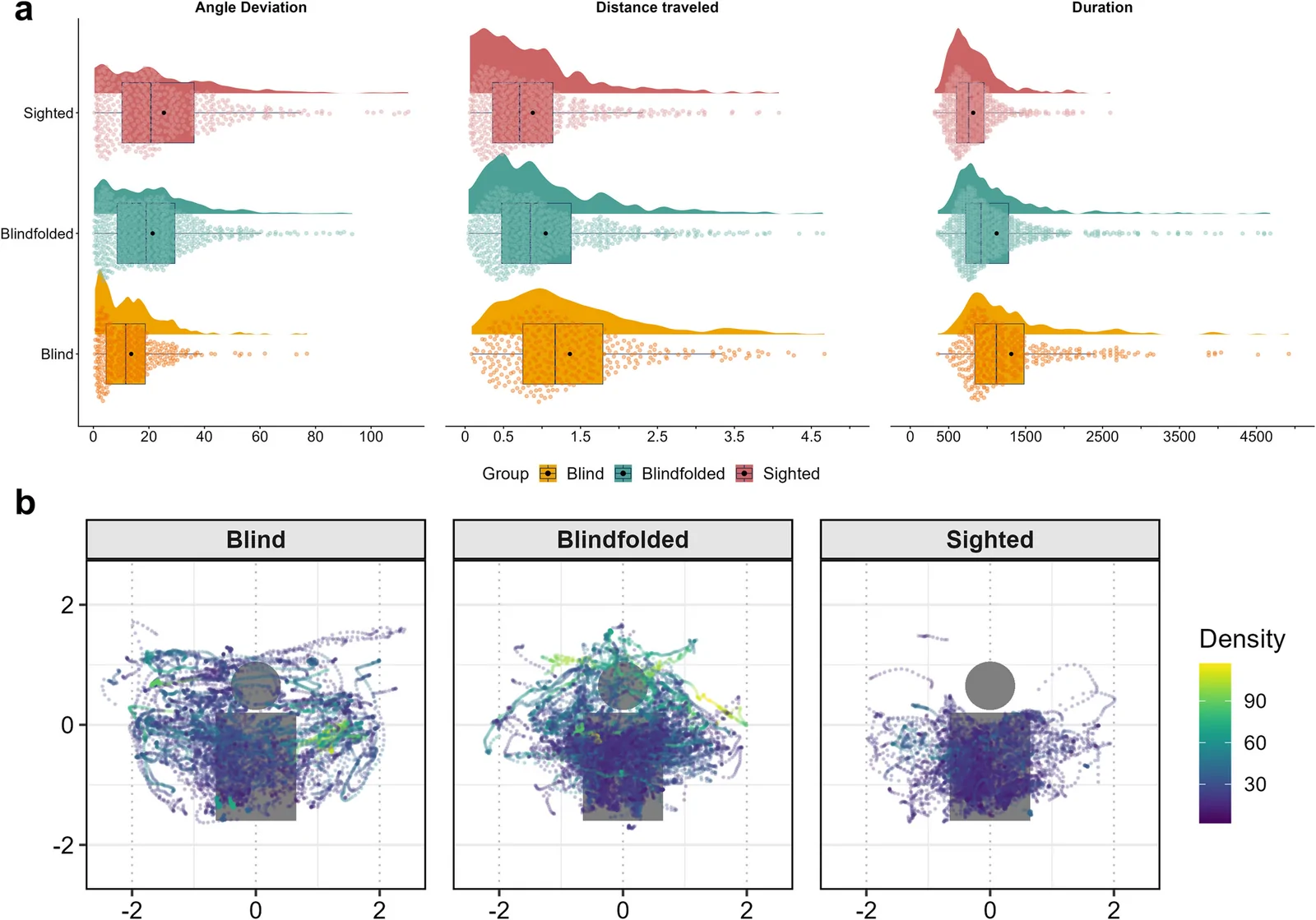
**(a)** Gesture precision (**left**; measured as angle deviation), gesture size (**center**; hand travel distance measured in unit of shoulder length, where 1 equals the speaker’s shoulder width), and gesture duration (**right**; in milliseconds). Smaller angle deviation values indicate better alignment between path gestures and event motion trajectories. **(b)** Heatmaps of gesture use in space. The gray cylinder and rectangular shapes represent the speakers’ heads and bodies, with their positions based on a normalized body

To evaluate how precisely speakers’ path gestures aligned with the spatial location of events, we ran an LMER model with the fixed factor of visual status using the log-transformed angle deviation as a dependent variable. The model revealed an effect of visual status, χ^2^ (2) = 13.37, *p =*.001. The difference between gestures and event locations was smaller in the blind group than in the sighted (*β* = −0.56, *SE* = 0.15, *t* = −3.69, *p* =.008) and blindfolded (*β* = −0.44, *SE* = 0.15, *t* = −2.97, *p* =.023) groups. There was no difference between blindfolded and sighted speakers, *β* = −0.12, *SE* = 0.13, *t* = −0.87, *p* =.74. On average, blind speakers’ path gestures deviated 9.5° from the event location, compared with 16.6° for sighted speakers and 14.8° for blindfolded speakers, indicating that blind speakers showed less deviation (7.1° and 5.3° less, respectively) and thus more precise alignment between path gestures and event locations, as expected (Fig. 2a, left).

### Gesture size and duration

After analyzing gesture precision, we also extracted kinematic features of gesture size and duration based on the wrist position of the gesturing hand. These measures served as a preliminary validation to ensure that any observed differences in gesture precision across groups aligned with differences in these kinematic features. Note that gesture size was measured as the distance traveled by the gesturing hand in units of shoulder length (see Methods for more details).

We ran separate LMER models for the log-transformed gesture size and duration as dependent variables with the fixed factor of visual status. For gesture size, the model revealed an effect of visual status, χ^2^ (2) = 8.64, *p =*.013. Blind speakers produced larger gestures than sighted speakers (*β* =.65, *SE* =.22, *t* = 2.95, *p* =.023), but not than blindfolded speakers (*β* =.45, *SE* =.22, *t* = 2.02, *p* =.17). There was no difference between blindfolded and sighted speakers, *β* =.20, *SE* =.20, *t* = 1.00, *p* =.74. On average, the hands of blind speakers traveled 1.03 shoulder lengths, nearly twice as far as the 0.54 shoulder lengths observed for sighted speakers, indicating longer movement paths of the gesturing hand (Fig. 2a, center). Because larger gestures (i.e., greater distance) typically take more time to execute, a correlation between size and duration was expected. Prior to fitting the model predicting gesture duration, we therefore assessed potential multicollinearity by examining the correlation between these variables and calculating variance inflation factors (VIFs) to quantify how much shared variance might inflate the estimates. We found a moderate correlation between size and duration (*r* = 0.64), consistent with this expectation. The VIFs for both predictors were 1.49, below commonly accepted thresholds (e.g., values below 5 are generally considered acceptable, whereas values above 10 indicate serious multicollinearity problems; Akinwande et al., 2015; O’brien, 2007). These results indicate that multicollinearity was not problematic, and we therefore retained both measures in the analysis. However, to isolate the fixed effect of visual status on gesture duration, gesture size was included as a predictor in the model, allowing us to account for its independent contribution to variation in duration.

For gesture duration, the model revealed a fixed effect of visual status, χ^2^ (2) = 15.40, *p* <.001, and a fixed effect of size, χ^2^ (1) = 517.7, *p* <.001. Blind speakers took more time to produce gestures than sighted speakers (*β* = 0.22, *SE* = 0.06, *t* = 3.42, *p* =.010), but not than blindfolded speakers (*β* = 0.01, *SE* = 0.06, *t* = 0.18, *p* =.98). However, blindfolded speakers took more time than sighted speakers, *β* = 0.21, *SE* =.06, *t* = 3.63, *p* =.002. After controlling for gesture size, blind speakers took on average 215 ms longer to produce a gesture than sighted speakers, while blindfolded speakers took 203 ms longer than sighted speakers (Fig. 2a, right).

We also visualized the use of space in path gestures by creating heatmaps that display each gesture’s motion trajectory by group (Fig. 2b). The density is concentrated around the center and frontal gesture space for sighted and blindfolded speakers, whereas it is more spread out for blind speakers in the semicircular space, aligning with the actual event locations (see also Fig. 1a).

Given that the heatmaps suggest that gestures in the blind group are more evenly distributed, whereas those in the sighted and blindfolded groups are more irregular, we conducted an exploratory analysis of movement variability by calculating the entropy of changes in the x- and y-coordinates. Here, entropy quantifies the unpredictability of wrist movement trajectories in two-dimensional (2D) space. Low entropy represents a gesture whose speed and direction are consistent or change smoothly in a predictable way; high entropy means irregular, hard‑to‑predict fluctuations in speed and/or direction from frame to frame.

The results showed that gesture entropy was significantly lower in the blind group than in both the blindfolded (*β* = −0.15, *SE* =.05, *t* = −3.32, *p* =.005) and the sighted groups (*β* = −0.15, *SE* =.05, *t* = −3.23, *p* =.006), with no difference between the latter two (*β* =.001, *SE* =.04, *t* =.03, *p* =.99). This indicates that gestures produced by blind participants are not only more spatially precise in relation to target events, but also show smoother coordination across spatial and temporal dimensions, reflected in more stable and predictable movement trajectories. Together, these findings point to a stronger integration between environmental representation and motor movements.

## Discussion

Our findings reveal that visuospatial experience influences the form features of gestures. Compared to sighted and blindfolded speakers, blind speakers produce spontaneous path-only gestures that more precisely follow the actual trajectory of motion events. This greater precision is accompanied by changes in gesture size and duration. Blind speakers use a broader range of space while gesturing, extending their hands within the semi-circular area around them. Thus, their path-only gestures reflect a more accurate mapping of spatial sounds to actual space.

These results are in line with findings from previous research showing blind people’s enhanced spatial localization abilities (Battal et al., 2020; Lessard et al., 1998; Lewald, 2013; Röder et al., 1999). Our findings, therefore, show that the altered spatial representations of blind people are also manifested in their gestural behavior during motion event descriptions. While language primarily determines the grammatical structure of co-speech gestures (Kita & Özyürek, 2003; Özçalışkan et al., 2016), the gesture kinematics are modulated by blind individuals’ sensorimotor experiences (see also Mamus et al., 2023, 2025). This provides compelling new evidence for theories highlighting the influence of sensorimotor experience in gesture production (Hostetter & Alibali, 2008).

Previous research shows that blind speakers’ verbal descriptions tend to be more segmented using landmarks than those of sighted speakers, reflecting the sequential nature of the perceptual information they rely on (Iverson, 1999; Iverson & Goldin-Meadow, 1997; Mamus et al., 2023). Furthermore, blind speakers often rely on an egocentric perspective when describing landmarks (Mamus et al., 2023). By showing that gestures can also reflect greater spatial localization abilities of blind people, our findings support theories proposing that spatial representations derived from language or perception share similar structural properties (Bryant, 1997; Landau & Jackendoff, 1993; Taylor & Tversky, 1992), and further extend these theories to include gestures accompanying spatial language.

Interestingly, blindfolded speakers produced gestures comparable in size to those of both blind and sighted speakers, yet still took longer than sighted speakers to produce them, even after controlling for gesture size. However, their gestures were less spatially accurate than those of blind speakers. These modulations in gesture size and duration may reflect the absence of visual feedback from the hands. Previous studies have also shown differences in the elevations of pointing when blindfolded versus when the eyes are open (Wnuczko & Kennedy, 2011). In addition, closing the eyes can modulate auditory attention, even in a darkened room, by reducing the dominance of vision (Wöstmann et al., 2020). Consistent with this, blindfolded speakers’ verbal descriptions of paths were more similar to those of blind speakers (Mamus et al., 2019, 2023). Taken together, these findings suggest that a temporary absence of vision can influence spatial event representations and gesture kinematics to some extent, but a lifetime lack of visual experience influences gesture precision beyond a temporary absence of vision.

Why do blind speakers show more precise spatial alignment in their gestures than non-blind speakers when describing spatial information? Is this only a byproduct of their enhanced auditory processing and spatial localization abilities, or does producing accurate gestures provide a functional advantage during communication? Although these questions remain open, one possibility is that blind speakers benefit from their enhanced spatial abilities through gestures, which may help them construct better or more easily accessible representations of spatial scenes. Gestures can support self-oriented cognitive functions, such as reducing cognitive load when organizing spatial information for speech (Kita et al., 2017). Thus, gestures may offer cognitive benefits for people such as helping them mentally simulate motion around themselves. Future research could explore this possibility.

Developing a new measure like this is not free of challenges; we encountered some that needed to be carefully addressed. To calculate precision, we relied on both finger direction and arm orientation, as people interpret pointing gestures based on both factors, especially when the gestures are not directed straight ahead (Krause & Herbort, 2023). However, there was inevitable variability both between individuals and within individuals in gesturing behavior, which made it more challenging to establish a universal rule for detecting the spatial angle of gestures that applies to all types of gestures (see also Krause & Herbort, 2024, for a discussion of a similar challenge regarding individual variation in gestures).

This study contributes to the expanding field of computer vision and motion-tracking tools in gesture research (Pouw et al., 2021; Trujillo, 2024; Trujillo et al., 2018, 2020) by analyzing the kinematics of spontaneous gestures produced by blind, blindfolded, and sighted speakers during event descriptions. In addition, our findings are relevant for researchers working to design better algorithms for gesture recognition, particularly for technological devices intended for blind users (Khanna et al., 2024). Our work provides valuable insights into the role of sensorimotor experiences in multimodal language production as well as spatial cognition and opens new avenues for advancing existing gesture theories.

## References

[CR1] Akinwande, M. O., Dikko, H. G., & Samson, A. (2015). Variance inflation factor: As a condition for the inclusion of suppressor variable(s) in regression analysis. *Open Journal of Statistics,**5*(7), 754–767. 10.4236/ojs.2015.57075

[CR2] Baayen, R. H., Davidson, D. J., & Bates, D. M. (2008). Mixed-effects modeling with crossed random effects for subjects and items. *Journal of Memory and Language, Special Issue: Emerging Data Analysis,**59*(4), 390–412. 10.1016/j.jml.2007.12.005

[CR3] Bates, D., Mächler, M., Bolker, B., & Walker, S. (2015). Fitting linear mixed-effects models using lme4. *Journal of Statistical Software,**67*(1), 1. 10.18637/jss.v067.i01

[CR4] Battal, C., Occelli, V., Bertonati, G., Falagiarda, F., & Collignon, O. (2020). General enhancement of spatial hearing in congenitally blind people. *Psychological Science,**31*(9), 1129–1139. 10.1177/0956797620935584 32846109

[CR5] Beilock, S. L., & Goldin-Meadow, S. (2010). Gesture changes thought by grounding it in action. *Psychological Science,**21*(11), 1605–1610. 10.1177/0956797610385353 20889932 PMC2978768

[CR6] Bryant, D. J. (1997). Representing space in language and perception. *Mind & Language,**12*(3–4), 239–264. 10.1111/j.1468-0017.1997.tb00073.x

[CR7] Cattaneo, Z., & Vecchi, T. (2011). *Blind vision: The neuroscience of visual impairment*. MIT Press. 10.7551/mitpress/9780262015035.001.0001

[CR8] Chu, M., & Hagoort, P. (2014). Synchronization of speech and gesture: Evidence for interaction in action. *Journal of Experimental Psychology: General,**143*(4), 1726–1741. 10.1037/a0036281 24635187

[CR9] Coluccia, E., Mammarella, I. C., & Cornoldi, C. (2009). Centred egocentric, decentred egocentric, and allocentric spatial representations in the peripersonal space of congenital total blindness. *Perception,**38*(5), 679–693. 10.1068/p5942 19662943

[CR10] Cook, S. W., & Tanenhaus, M. K. (2009). Embodied communication: Speakers’ gestures affect listeners’ actions. *Cognition,**113*(1), 98–104. 10.1016/j.cognition.2009.06.006 19682672 PMC2763957

[CR11] Dufour, A., Després, O., & Candas, V. (2005). Enhanced sensitivity to echo cues in blind subjects. *Experimental Brain Research,**165*(4), 515–519. 10.1007/s00221-005-2329-3 15991030

[CR12] Gullberg, M., Hendriks, H., & Hickmann, M. (2008). Learning to talk and gesture about motion in French. *First Language,**28*(2), 200–236. 10.1177/0142723707088074

[CR13] Hostetter, A. B., & Alibali, M. W. (2008). Visible embodiment: Gestures as simulated action. *Psychonomic Bulletin & Review,**15*(3), 495–514. 10.3758/PBR.15.3.495 18567247

[CR14] Iachini, T., Ruggiero, G., & Ruotolo, F. (2014). Does blindness affect egocentric and allocentric frames of reference in small and large scale spaces? *Behavioural Brain Research,**273*, 73–81. 10.1016/j.bbr.2014.07.032 25078290

[CR15] Iverson, J. M. (1999). How to get to the cafeteria: Gesture and speech in blind and sighted children’s spatial descriptions. *Developmental Psychology,**35*(4), 1132–1142. 10.1037/0012-1649.35.4.1132 10442881

[CR16] Iverson, J. M., & Goldin-Meadow, S. (1997). What’s communication got to do with it? Gesture in children blind from birth. *Developmental Psychology,**33*(3), 453–467. 10.1037/0012-1649.33.3.453 9149924

[CR17] Khanna, P., Ramakrishnan, I., Jain, S., Bi, X., & Balasubramanian, A. (2024). Hand gesture recognition for blind users by Tracking 3D gesture trajectory. *Proceedings of the 2024 CHI Conference on Human Factors in Computing Systems, CHI ’24* (pp. 1–15). ACM. 10.1145/3613904.3642602 PMC11707651 39781365

[CR18] Kita, S., & Özyürek, A. (2003). What does cross-linguistic variation in semantic coordination of speech and gesture reveal?: Evidence for an interface representation of spatial thinking and speaking. *Journal of Memory and Language,**48*(1), 16–32. 10.1016/S0749-596X(02)00505-3

[CR19] Kita, S., Alibali, M. W., & Chu, M. (2017). How do gestures influence thinking and speaking? The gesture-for-conceptualization hypothesis. *Psychological Review,**124*(3), 245–266. 10.1037/rev0000059 28240923

[CR20] Krause, L.-M., & Herbort, O. (2023). Just visual context or part of the gesture? The role of arm orientation in bent pointing interpretation. *Acta Psychologica,**241*, 104062. 10.1016/j.actpsy.2023.104062 39491420

[CR21] Krause, L.-M., & Herbort, O. (2024). Perception of pointing gestures in 3D space. *Scientific Reports,**14*(1), 27595. 10.1038/s41598-024-78129-4 39528588 PMC11554782

[CR22] Kuznetsova, A., Brockhoff, P. B., & Christensen, R. H. B. (2017). lmerTest package: Tests in linear mixed effects models. *Journal of Statistical Software,**82*(1), 1. 10.18637/jss.v082.i13

[CR23] Landau, B., & Jackendoff, R. (1993). Whence and whither in spatial language and spatial cognition? *Behavioral and Brain Sciences,**16*(2), 255–265. 10.1017/S0140525X00029927

[CR24] Lenth, R. (2022). *Emmeans: Estimated marginal means, aka least-squares means.* R package version 1.7.3. Retrieved March 25, 2026, from https://CRAN.R-project.org/package=emmeans

[CR25] Lessard, N., Paré, M., Lepore, F., & Lassonde, M. (1998). Early-blind human subjects localize sound sources better than sighted subjects. *Nature,**395*(6699), 278–280. 10.1038/26228 9751055

[CR26] Lewald, J. (2013). Exceptional ability of blind humans to hear sound motion: Implications for the emergence of auditory space. *Neuropsychologia,**51*(1), 181–186. 10.1016/j.neuropsychologia.2012.11.017 23178211

[CR27] Mamus, E., Rissman, L., Majid, A., & Özyürek, A. (2019). Effects of blindfolding on verbal and gestural expression of path in auditory motion events. In A. K. Goel, C. M. Seifert, & C. C. Freksa (Eds.), *Proceedings of the 41st Annual Meeting of the Cognitive Science Society (CogSci 2019)* (pp. 2275–2281). Cognitive Science Society.

[CR28] Mamus, E., Speed, L. J., Rissman, L., Majid, A., & Özyürek, A. (2023). Lack of visual experience affects multimodal language production: Evidence from congenitally blind and sighted people. *Cognitive Science,**47*(1), e13228. 10.1111/cogs.13228 36607157 PMC10078191

[CR29] Mamus, E., Speed, L. J., Ortega, G., Majid, A., & Özyürek, A. (2025). Gestural and Verbal Evidence of Conceptual Representation Differences in Blind and Sighted Individuals. *Cognitive Science,**49*(10), e70125. 10.1111/cogs.70125 41082278 PMC12517398

[CR30] McNeill, D. (1992). *Hand and mind: What gestures reveal about thought*. University of Chicago Press.

[CR31] Nilsson, M. E., & Schenkman, B. N. (2016). Blind people are more sensitive than sighted people to binaural sound-location cues, particularly inter-aural level differences. *Hearing Research,**332*, 223–232. 10.1016/j.heares.2015.09.012 26433052

[CR32] O’brien, R. M. (2007). A caution regarding rules of thumb for variance inflation factors. *Quality & Quantity,**41*(5), 673–690. 10.1007/s11135-006-9018-6

[CR33] Özçalışkan, Ş, Lucero, C., & Goldin-Meadow, S. (2016). Is seeing gesture necessary to gesture like a native speaker? *Psychological Science,**27*(5), 737–747. 10.1177/0956797616629931 26980154

[CR34] Özçalışkan, Ş, Lucero, C., & Goldin-Meadow, S. (2018). Blind speakers show language-specific patterns in co-speech gesture but not silent gesture. *Cognitive Science,**42*(3), 1001–1014. 10.1111/cogs.12502 28481418

[CR35] Pasqualotto, A., Spiller, M. J., Jansari, A. S., & Proulx, M. J. (2013). Visual experience facilitates allocentric spatial representation. *Behavioural Brain Research,**236*, 175–179. 10.1016/j.bbr.2012.08.042 22960256

[CR36] Pouw, W., & Trujillo, J. (2021). *Body Tracking Using MediaPipe* [Computer software]. Retrieved November 15, 2024, from https://github.com/WimPouw/EnvisionBootcamp2021/blob/main/Python/BodyTracking_MediaPipe

[CR37] Pouw, W., Wassenburg, S. I., Hostetter, A. B., de Koning, B. B., & Paas, F. (2020). Does gesture strengthen sensorimotor knowledge of objects? The case of the size-weight illusion. *Psychological Research,**84*(4), 966–980. 10.1007/s00426-018-1128-y 30552506 PMC7239830

[CR38] Pouw, W., Dingemanse, M., Motamedi, Y., & Özyürek, A. (2021). A systematic investigation of gesture kinematics in evolving manual languages in the lab. *Cognitive Science,**45*(7), e13014. 10.1111/cogs.13014 34288069 PMC8365719

[CR39] R Core Team. (2025). *R: A language and environment for statistical computing*.

[CR40] Röder, B., Teder-Sälejärvi, W., Sterr, A., Rösler, F., Hillyard, S. A., & Neville, H. J. (1999). Improved auditory spatial tuning in blind humans. *Nature,**400*(6740), 162–166. 10.1038/22106 10408442

[CR41] Ruggiero, G., Ruotolo, F., & Iachini, T. (2021). How ageing and blindness affect egocentric and allocentric spatial memory. *Quarterly Journal of Experimental Psychology*, 17470218211056772. 10.1177/17470218211056772 34670454

[CR42] Slobin, D. (1996). From “thought” and “language” to “thinking for speaking.” In J. J. Gumperz & S. C. Levinson (Eds.), *Rethinking linguistic relativity* (pp. 70–96). Cambridge University Press.

[CR43] Talmy, L. (1983). How language structures space. In H. L. Pick & L. P. Acredolo (Eds.), *Spatial orientation* (pp. 225–282). Springer US. 10.1007/978-1-4615-9325-6_11

[CR44] Talmy, L. (1985). Lexicalization patterns: Semantic structure in lexical forms. In T. Shopen (Ed.), *Language typology and semantic description* (pp. 36–149). Cambridge University Press.

[CR45] Taylor, H. A., & Tversky, B. (1992). Spatial mental models derived from survey and route descriptions. *Journal of Memory and Language,**31*(2), 261–292. 10.1016/0749-596X(92)90014-O

[CR46] Thinus-Blanc, C., & Gaunet, F. (1997). Representation of space in blind persons: Vision as a spatial sense? *Psychological Bulletin,**121*(1), 20–42. 10.1037/0033-2909.121.1.20 9064698

[CR47] Trujillo, J. P. (2024). Motion-tracking technology for the study of gesture. *The Cambridge handbook of gesture studies. *Cambridge University Press. https://pure.mpg.de/pubman/faces/ViewItemOverviewPage.jsp?itemId=item_3587978

[CR48] Trujillo, J. P., Simanova, I., Bekkering, H., & Özyürek, A. (2018). Communicative intent modulates production and comprehension of actions and gestures: A Kinect study. *Cognition,**180*, 38–51. 10.1016/j.cognition.2018.04.003 29981967

[CR49] Trujillo, J. P., Simanova, I., Bekkering, H., & Özyürek, A. (2020). The communicative advantage: How kinematic signaling supports semantic comprehension. *Psychological Research,**84*(7), 1897–1911. 10.1007/s00426-019-01198-y 31079227 PMC7772160

[CR50] Trujillo, J. P., Özyürek, A., Holler, J., & Drijvers, L. (2021). Speakers exhibit a multimodal Lombard effect in noise. *Scientific Reports,**11*(1), 16721. 10.1038/s41598-021-95791-0 34408178 PMC8373897

[CR51] Ünal, E., Manhardt, F., & Özyürek, A. (2022). Speaking and gesturing guide event perception during message conceptualization: Evidence from eye movements. *Cognition, 225*, 105127. 10.1016/j.cognition.2022.105127 35617850

[CR52] Ünal, E., Mamus, E., & Özyürek, A. (2024). Multimodal encoding of motion events in speech, gesture and cognition. *Language and Cognition,**16*(4), 785–804. 10.1017/langcog.2023.61

[CR53] Wnuczko, M., & Kennedy, J. M. (2011). Pivots for pointing: Visually-monitored pointing has higher arm elevations than pointing blindfolded. *Journal of Experimental Psychology: Human Perception and Performance,**37*(5), 1485–1491. 10.1037/a0024232 21688946

[CR54] Wöstmann, M., Schmitt, L.-M., & Obleser, J. (2020). Does closing the eyes enhance auditory attention? Eye closure increases attentional alpha-power modulation but not listening performance. *Journal of Cognitive Neuroscience,**32*(2), 212–225. 10.1162/jocn_a_01403 30912726

